# Polymer Membranes as Innovative Means of Quality Restoring for Wastewater Bearing Heavy Metals

**DOI:** 10.3390/membranes12121179

**Published:** 2022-11-23

**Authors:** Teodor Sandu, Andrei Sârbu, Simona Căprărescu, Elena-Bianca Stoica, Tanța-Verona Iordache, Anita-Laura Chiriac

**Affiliations:** 1 Advanced Polymer Materials and Polymer Recycling Group, National Institute for Research & Development in Chemistry and Petrochemistry ICECHIM, Spl. Independentei 202, 6th District, 060021 Bucharest, Romania; 2 Department of Inorganic Chemistry, Physical Chemistry and Electrochemistry, Faculty of Chemical Engineering and Biotechnologies, University Politehnica of Bucharest, Ghe. Polizu Street, No. 1-7, 011061 Bucharest, Romania

**Keywords:** polymers, polymer blends, tuneable porosity, membranes, heavy metals

## Abstract

The problem that has aroused the interest of this review refers to the harmful effect of heavy metals on water sources due to industrial development. In this respect, the review is aimed at achieving a literature survey on the outstanding results and advancements in membranes and membrane technologies for the advanced treatment of heavy metal-loaded wastewaters. Particular attention is given to synthetic polymer membranes, for which the proper choice of precursor material can provide cost benefits while ensuring good decontamination activity. Furthermore, it was also found that better removal efficiencies of heavy metals are achieved by combining the membrane properties with the adsorption properties of inorganic powders. The membrane processes of interest from the perspective of industrial applications are also discussed. A noteworthy conclusion is the fact that the main differences between membranes, which refer mainly to the definition and density of the pore structure, are the prime factors that affect the separation process of heavy metals. Literature studies reveal that applying UF/MF approaches prior to RO leads to a better purification performance.

## 1. Introduction

Water plays a fundamental role for humans, living organisms and, implicitly, for the economy. To state it briefly: “Water means life!”. Unfortunately, we are currently facing serious problems related to water pollution. Both developed and developing countries deal with water quality issues primarily due to anthropogenic activities. Industrial development around the world has had the effect of releasing substances that are toxic to the environment in general and water in particular. In this context, it is worth noting that various branches of industry, such as metal plating plants, mining operations, and tanneries, yield Heavy Metals (HMs). Certain HMs, such as arsenic (As), lead (Pb), mercury (Hg), cadmium (Cd), chromium (Cr), copper (Cu), nickel (Ni), selenium (Se), and zinc (Zn), naturally occur in the earth’s crust. Some of them are also known as trace elements. HMs are among the most persistent pollutants in wastewater [1,2,3,4]. Therefore, a significant number of studies have been devoted to identifying viable means of restoring water quality. An HM is any chemical element with a relatively high density, which is toxic or poisonous even at low concentrations. There is much controversy as to the density at which a metal can be considered a heavy metal. According to some authors, the density of HMs should be greater than 4 ± 1 g/cm^3^, whereas others consider that density should exceed 5 g/cm^3^ or even 6 g/cm^3^. Although not strictly defined, many contaminants of terrestrial and freshwater ecosystems are traditionally called heavy metals [5,6,7,8]. None of these HMs are biodegradable; their effects on humans differ not only from one metal to another, but even for the same metal, in which case the effects are different depending on the amount ingested. Thus, some HMs, such as copper, selenium, and zinc, are beneficial when ingested in small amounts, as they play the role of trace elements involved in human metabolism [9]. However, the situation changes at higher concentrations, leading to poisoning. The worse scenario due to HMs bioaccumulation is represented by an increase in the metal concentration in the body compared to its concentration in the environment. For instance, the accumulation of HMs in plant tissues can lead to toxicity because it inhibits the growth of roots and shoots [10,11,12,13]. Likewise, HMs can also enter aquatic organisms, thus reaching the food chain with consequences for animal and human health. HMs can directly enter the body through drinking water and inhaled air, posing toxicity issues as they interfere with the body’s biochemical reactions. Ecotoxic issues related to HMs refer to the side effects when consumed beyond the biological limits [5]. The harmful effects of HMs in cases of exceeding the permissible limits are somewhat different, and some examples are presented below. Arsenic exposure affects the cardiovascular, dermatological, nervous, hepatobiliary, renal, gastrointestinal, and respiratory systems. Antimony provokes nausea, vomiting, and diarrhoea. Cadmium is associated with kidney dysfunction as well as lung conditions. Chromium can provoke kidney and liver damage, affecting both circulatory and nervous tissue. Lead has negative effects on haemoglobin synthesis, affecting the kidneys, gastrointestinal tract, and joints, as well as the reproductive and nervous systems. Mercury, a toxic substance with no role in human biochemistry or physiology, has deleterious effects as it may cause tremors, gingivitis, and/or minor psychological changes, as well as miscarriage and birth defects. In small amounts, nickel is needed by the human body to produce red blood cells, but exceeding this amount can lead to weight loss, heart and liver damage, and skin irritation. Selenium is only needed in small amounts in humans and animals, otherwise it can damage the nervous system, causing fatigue and irritability [5,8,14].

The present review, dedicated to the use of a membrane for removing heavy metals from wastewater, intends to systematize the literature data in the domain, which is useful for specialists working on membrane preparation and their application in the field of environmental protection.

The environment is a dynamic system quantified by various factors (like pH, pressure, temperature, and human activity). Humans generate new substances in the environment following their activity, with considerable effects on the ecosystem, disrupting the initial functions of both the human organism and other living organisms. In this respect, the use of polymer membranes in wastewater treatment is advantageous for several reasons, referring to the production of high-quality water with a high recovery efficiency of heavy metals and fair implementation costs. The research on the use of polymer membranes to mitigate the risk of environmental pollution is undergoing constant development due to the possibility of using this technology to purify different types of wastewaters as well as the air and the soil, which is particularly important from the environmental protection viewpoint.

## 2. Conventional Technologies for Heavy Metals (HMs) Removal from Wastewaters

**Figure 1:**
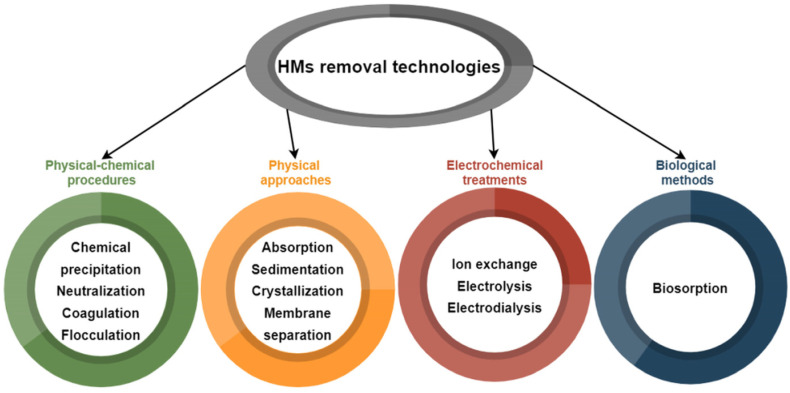
Conventional membrane technologies used for HMs removal from wastewaters.

The methods involved in HMs removal may be classified as follows: physical–chemical procedures, e.g., chemical precipitation, neutralization, coagulation, flocculation; physical approaches, such as absorption, sedimentation, crystallization, and membrane separation; electrochemical treatments using ion exchange, electrolysis, or electrodialysis; and biological methods, mainly biosorption, as shown in Figure 1 [15].

Physical–chemical and physical methods include mechanical screening, hydrodynamic classification, gravity concentration, flotation, magnetic separation, electrostatic separation, and attrition washing [16]. Chemical precipitation relies on turning heavy metals into their derivatives (hydroxide, sulphide, carbonate, and phosphate), which can subsequently precipitate [17].

Coagulation flocculation processes take the zeta potential values into account as a measure of the interactions between the pollutant and coagulant/flocculant [15]. Electrochemical processes, i.e., electrolysis, involve the passage of electricity through an aqueous solution of metal(s) [18]. Either weak acidic compounds or hydroxides are also used to promote coagulation. Ion exchange processes bring along cost benefits since low-cost materials may be considered [17]. Nevertheless, each method has advantages and disadvantages [13,19,20,21,22,23]. For example, the main disadvantage of precipitation processes is the high consumption of chemical reagents that reduce metals to an acceptable level for discharge. On the other hand, adsorption is more effective and versatile for removing heavy metals. Yet, following multiple adsorption–desorption metal recovery steps, a problem of slag management arises [13,24,25]. It can also be noted that all the previous mentioned approaches pose energy issues and fail when it comes to achieving an advanced removal, as compared to membrane processes [26].

Membranes act as interfaces between two phases, regulating the transport of matter as a result of the differences in pressure, temperature, concentration, and electric potential, being thus often employed as viable means of filtering and cleaning [27]. Within the membrane processes, not all components are transported at the same rate, and thus separation occurs. The use of polymeric membranes for the removal of heavy metals shows certain benefits, the most relevant being low energy consumption, high efficiency, and mild operating conditions [28]. Therefore, polymer membranes may be regarded as an alternative to traditional processes used for HMs removal [28,29,30].

**Figure 2:**
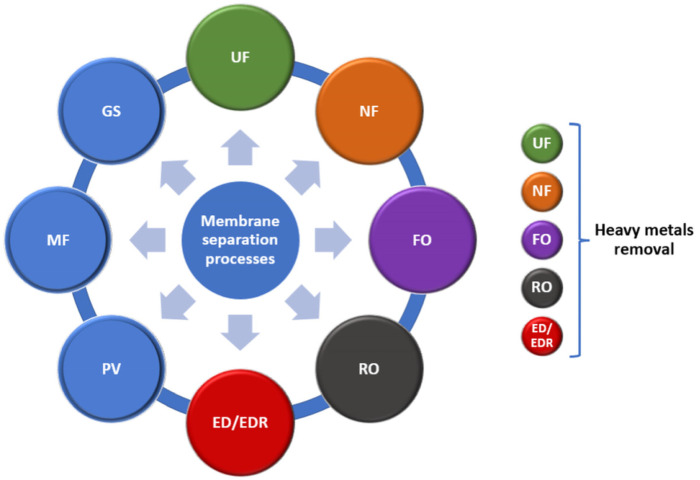
Separation processes carried out by membrane processes, with emphasis on those involved in heavy metals removal.

Membrane separation and adsorption processes are of great interest for treating wastewater loaded with heavy metals [22,25,31]. Membrane technologies show certain technological benefits over other conventional approaches. However, price and feasibility concerns limit the application of membranes to a certain extent [4]. Membrane separation processes may be divided into several classes: (1) ultrafiltration (UF); (2) nanofiltration (NF); (3) forward osmosis (FO); (4) reverse osmosis (RO); (5) electrodialysis/electrodialysis reversal (ED/EDR); (6) pervaporation (PV); (7) microfiltration (MF); and (8) gas separation (GS), as shown in Figure 2. Of these eight processes, UF, NF, FO, ED/EDR, and RO are in use for decontaminating waters containing various types of pollutants (organic and inorganic compounds, solid materials). In this context, it is worth mentioning their application to the removal of heavy metals [32,33,34]. The membrane approaches can be used not only individually, but in a combined manner. In this respect, Jiang et al. approached a RO–ED–BMED (bipolar membrane electrodialysis) sequence for the amendment of cold-rolling wastewater [35].

Because the use of membranes is not only modern, but also has the advantage of high flexibility and properties control by adjusting the parameters of their preparation, this review describes only the advancements in membrane technologies [36,37]. For developing commercial membranes, polysulfone, polyacrylonitrile, polyvinylidene fluoride, polypropylene (PP), polyamide, and polyethylene (PE) are frequently used [38,39]. The choice is mainly dictated by the chemical stability of the polymer and by the nature of functional groups in the backbone [37]. Therefore, synthetic polymer-based membranes are preferred due to the possibility of ensuring a specific morphology [37]. Tuning the membrane’s morphology to deliver the desired transport properties is critical, which is why this has been the subject of many critical reviews [13,37,40,41,42,43,44]. Furthermore, synthetic polymers also offer flexibility to the membrane preparation process, giving the possibility to choose between one polymer and another, or a mixture of polymers instead of a single polymer, all to maximize the efficiency of the final membrane. Hence, special attention should be paid to the membrane preparation procedure in order to be able to customize them for a targeted application. 

**Figure 3:**
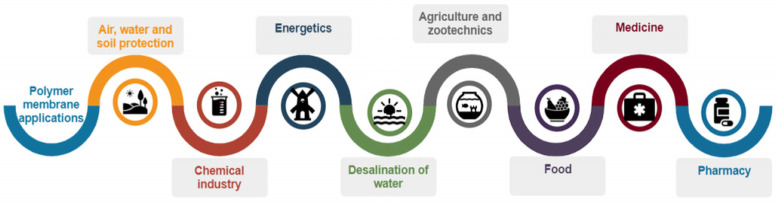
A schematic representation of polymer membrane applications.

The functionalities of polymer membranes allow their application in various fields, as shown in Figure 3.

The most frequently applied procedures for preparing membranes are phase inversion, interfacial polymerization, composite coating, multilayer composite casting, sintering, stretching, track etching, and electrospinning [42,45,46,47,48,49]. Thereby, together with innovations in membrane processes, the efficiency of emerging membrane types will also be discussed with respect to the preparation procedure. 

## 3. Types of Membranes for Water Quality Restoration

Given the multitude of membranes used in water reconditioning, a classification in this respect becomes necessary. According to the literature, several classification criteria are known as follows: nature of the precursors, morphology, geometry, operating regime, type of membrane process, and cross section. The latter criterion divides membranes into anisotropic (asymmetric) and isotropic (symmetric), respectively [42]. 

### 3.1. Membranes Based on Different Types of Precursors

In terms of the nature of the precursors used, there are three major classes: organic, inorganic, and mixtures thereof [44]. Among the most widely used materials, ceramic materials (Al_2_O_3_, TiO_2_, ZrO_2_, SiO_2_, TiO_2_-SiO_2_, TiO_2_-ZrO_2_, Al_2_O_3_-SiC), graphene, and carbon nanotubes are preferred as inorganic precursors [50,51,52,53,54,55,56,57], while polymers are of main interest as organic precursors. Membrane-precursor polymers include polyvinyl alcohol (PVA), polyimide (PI), polypropylene (PP), polyethersulfone (PES), cellulose acetate (CA), cellulose nitrate (CN), polysulfone (PS), polyvinylidene fluoride (PVDF), polyacrylonitrile (PAN), polyurethane (PU), and poly (tetrafluoroethylene) (PTFE, also known as Teflon) [58,59,60,61,62,63,64,65,66,67]. Other used polymers include Polyamide 6 (PA 6), polyethersulfone (PES), and polyvinylchloride (PVC). PAN may be also used together with other modifiers [68]. Despite the advantages of PAN, an important deficiency which limits its use for developing membranes is that it is prone to fouling [69]. Siekerka et al. developed membranes with affinity towards cobalt by incorporating 5-chloro-8-hydroxyquinoline within a PAN membrane. This membrane proved to be useful for recovering cobalt from lithium, cobalt, and nickel solutions [68].

The use of hybrid precursors of the inorganic–organic type involves the introduction of inorganic materials in a system of polymeric materials. Although phase separation membranes have a homogeneous chemical composition, some differences in terms of their structure, pore size, porosity, and thickness are present in bulk [70]. Thin film composite membranes can be considered phase separation membranes. They have a typical morphology, which consists of a thin top layer and a thick, porous polymer material support. Furthermore, macroporous membranes, also known as screening membranes, are those membranes for which the diameter of their pores can vary from 0.1 to 5 µm and for which the filtration process relies on the difference between particle size and the membrane pore size [71,72].

### 3.2. Membranes Based on Porosity Feature

**Figure 4:**
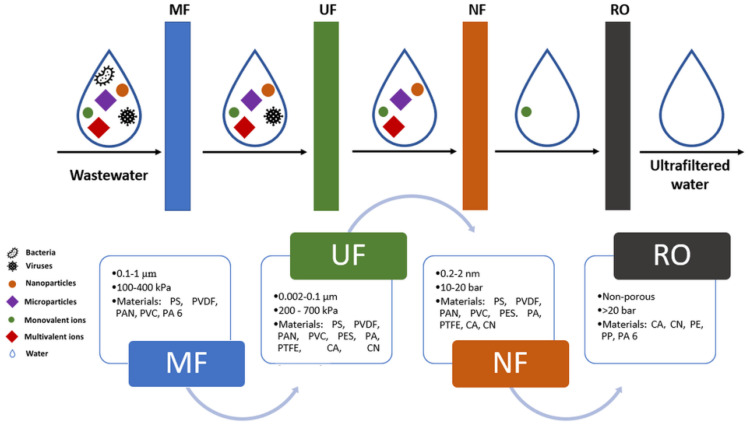
Membrane separation processes and polymers used for this purpose.

The most widely used classification differentiates membranes as porous and non-porous. Non-porous membranes are useful for GS and PV, as well as in some osmosis and dialysis processes (Figure 4). At the same time, the solution transfer is closely related to the transport rate [73]. Porous membranes perform MF, UF, and NF processes (Figure 4). In other words, using non-porous film membranes involves the application of a driving force, such as pressure, concentration, or electric field gradients, to determine the transport of the dissolved substance.

Electrically charged membranes, also known as ion exchange membranes, are made of either dense, non-porous films or microporous structures. These membranes have modified surfaces by means of negative or positive ions and often have anisotropic structures. The factors influencing the transport mechanism in such membranes are the solute ion concentration and the charge density [74]. Anisotropic membranes are mainly used to treat heavy metals in wastewater. Also, membranes used for water treatment purposes can be divided into flat membranes and hollow fibre membranes [75]. Flat membranes are preferred due to the lower costs.

The membranes may be prepared by melt processing (for instance, PE, PP, PTFE, and PA) or from a polymer solution (for example, PS, PAN, and PVC). Membranes are conventionally prepared from a polymer solution by phase inversion, which refers to a transformation of the polymer from a liquid phase to a solid phase under controlled conditions [73,76]. This transformation can be achieved through three approaches: precipitation by controlled evaporation; thermally induced phase separation; and immersion precipitation. Precipitation by controlled evaporation, also known as dry phase inversion, involves dissolving the polymer in a highly volatile solvent. The polymer precipitates during solvent evaporation, forming a skinned membrane (thin layer). In the thermally induced phase separation, the precipitation of the polymer in the solution is determined by the changes in the polymer’s solubility due to increasing or decreasing solution temperature. Immersion precipitation occurs by extruding a polymer solution in a coagulation bath, which contains a non-solvent for the polymer. A polymer film is formed as the solvent passes from the polymer solution into the coagulation bath. This method is also called wet phase inversion or non-solvent induced phase separation (NIPS).

Out of all the classification criteria of membranes mentioned previously, the most important one takes into consideration the type of membrane process (as depict in Figure 4). For this reason, the following sections provide a detailed description of each process with emphasis on the process parameters and membrane nature.

## 4. Membrane Approaches for Water Decontamination

### 4.1. Ultrafiltration (UF)

UF is a very effective membrane technique when it comes to the removal of dissolved and colloidal matter, and also has the advantage of performing at low transmembrane pressures. UF uses a permeable membrane in order to separate heavy metals, macromolecules, and suspended solids from the inorganic solution, a phenomenon based on the pore size (usually 5–20 nm) and the molecular weight of the separation compounds (10,000–100,000 Da). Out of a desire for high efficiency of metal ion removal, Micellar Enhanced Ultrafiltration (MEUF) and Polymer Enhanced Ultrafiltration (PEUF) were investigated. The concept of MEUF was introduced by Dunn et al. [77] in the 1980s, and was applied to remove dissolved organic compounds and multivalent metal ions. Subsequently, MEUF has proven effective for removing metal ions from wastewater and involves the addition of surfactants to wastewater. When the concentration of surfactants in aqueous solutions exceeds the Critical Micellar Concentration (CMC), the surfactant molecules are organized into micelles, capable of binding metal ions to form large metal-surfactant structures. Micelles loaded with metal ions can be retained by a UF membrane with smaller pores than the size of the micelles, while non-entrapped species are allowed to pass through the UF membrane. For proper retention efficiency, surfactants with an electrical charge opposite of that of the targeted ions should be considered. In this context, it is worth mentioning that Sodium Dodecyl Sulphate (SDS) is an anionic surfactant often used to efficiently remove HM ions by MEUF. The efficiency of metal removal by MEUF depends on the characteristics and concentrations of metals and surfactants, the pH of the solution, and the ionic strength, as well as other membrane performance parameters.

Landaburu-Aguirre et al. studied zinc removal from synthetic wastewater by MEUF using SDS, in which case values up to 99% were recorded for the rejection coefficients when the molar ratio of surfactant to metal (S/M) exceeded 5 [78]. Apart from Ni (II), unexpected metal retention of over 90% was recorded for initial SDS concentrations lower than CMC. The retentate phase consisted of a mixture of surfactants and heavy metals retained by the membrane. Given the importance of the surfactant for operating costs, special interest has been paid to the possibility of reusing it. In this context, surfactant and heavy metals should be removed to avoid the risk of secondary pollution.

Li and collab. approached a chelation-UF-acidification-UF sequence to separate Cd (II) or Zn (II) from SDS mycelium in a simulated MEUF retentate solution while reusing SDS [79]. With the SDS recovered in the MEUF process, the removal efficiency for Cd (II) and Zn (II) were 90.3% and 89.6%, respectively. The use of acidic agent H_2_SO_4_ at pH 1.0 led to the highest separation of heavy metal ions (98.0% for Cd (II), 96.1% for Zn (II)), while also allowing the recovery of SDS (58.1% for Cd (II), 54.3% for Zn (II)). The recovered SDS efficiency was 88.1% for Cd (II) removal and 87.8% for Zn (II) removal in MEUF.

Literature studies report the use of UF membranes, both organic and ceramic, in order to decontaminate aqueous solutions. The integration of complexing agents or charged electrolytes in membranes is beneficial in terms of improving their performance [80,81,82]. The Bruening Group studied the process of cationic metals removal by approaching NF, carried out using polyelectrolyte-modified alumina membranes [83]. Most of the ceramic and inorganic membranes have a tubular configuration. Research has been conducted on the use of inorganic membranes in Cr (VI) removal. Pugazhenthi et al. prepared a carbon-supported ultrafiltration membrane modified by gas-phase nitration (using NOx) and amination (using hydrazine hydrate), further used to separate Cr (VI) from an aqueous solution [84]. The effective pore radius of the unchanged carbon, nitrate, and amine membranes was 2.0, 2.8, and 3.3 nm, respectively. However, it has been found that the water flow of the modified membrane increases twice as much as that of the unmodified membrane. When separating the chromic acid solution, all three types of carbon membranes were tested. The following degrees of rejection were recorded: 96% for the unmodified membrane, 88% for the aminated membrane, and 84% for the nitrated membrane, respectively.

Another important direction of research was quantifying the effect of membrane modification with chitosan in a study focusing on the removal of copper and zinc ions [85]. The cellulose generated by Amicon (YM10) was used as an ultrafilter, with a rejection rate of approximately 100% for Cu (II) and 95% for Zn (II) ions (at a pH between 8.5 and 9.5). The use of chitosan substantially increased (6–10 times) the purification efficiency, which may be due to the coordination processes involved in the amino groups of chitosan. When working at acidic pH, these functional groups are protonated. Most chelating processes involve the formation of donor–acceptor interactions as a result of the attachment of metal ions to the functional groups of the chelating agent [85].

Saffaj et al. studied the process of removing Cd (II) and Cr (III) ions from a synthetic solution using UF membranes prepared using inexpensive raw materials, ZnAl_2_O_4_—TiO_2_ type, with high rejection rates of 93% Cd (II) and 86% Cr (III), respectively [86]. These satisfactory results are due to the strong interactions established between the divalent cations and the positive charge of the membranes. Depending on the membrane characteristics, UF can lead to a removal efficiency of over 90% at a metal concentration of 10 to 112 mg/L, for a pH value between 5 and 9.5 and at 2–5 pressure bars [87], which involves significant operating costs. In UF systems, fouling is a major problem. However, it may be solved by tunning the membrane hydrophilicity [88] using hydrophilic polymers, such as polyvinylpyrrolidone (PVP) and polyethylene glycol (PEG). Polyether sulfone (PES) carries repeated ether and sulfone bonds, alternating between aromatic rings, which also endow the membrane with enhanced mechanical stability. For this reason, PES is a membrane material of great interest, but its use is somewhat limited by hydrophobicity, a deficiency that can be overcome by blending with other hydrophilic polymers, such as cellulose acetate phthalate. Moreover, PES is unsuitable for harsh cleaning because of its low oxidation and chemical resistances. However, its modification with cellulose acetate represents a viable solution to solve these problems as well [89,90].

Polymeric membranes prepared by non-solvent induced phase separation (NIPS) dominate commercially available UF membranes. Among them, polysulfone (PS) and polyethersulfone (PES) UF membranes are widely manufactured for industrial applications. Nevertheless, the hydrophobicity of those materials can cause serious fouling issues requiring chemical modification measures. Polymeric additives, often hydrophilic polymers, can be added to the casting solution for better control over porosity. These additives do not play only a pore-forming agent role, but they suppress macro void formation. Polyvinylpyrrolidone (PVP) and poly (ethylene glycol) (PEG) have been intensively used as additives during the preparation of PES UF membranes by phase inversion. 

Literature studies describe in detail the formation mechanism of PES–PVP membranes. Adding PVP in the PES–NMP (N-methyl-2-pyrrolidone) casting solution suppressed the formation of macrovoids, whereas the characteristics and performance of PES–PVP blend membranes were influenced by the concentration and the molar mass of PVP [91,92,93]. The highest product permeation rate, examined using a PEG solution, corresponded to the unit PVP/PES weight ratio [94]. The retention coefficient, as well as the surface roughness, increased with increasing molar masses of PVP. Therefore, the use of PVP inhibited the fouling effect to some extent. PEG with various molar masses was also added during the preparation of PES UF membranes [95], in which case it was found that the performance of the resultant membranes was highly dependent on the PEG concentration and its molar mass. Membranes prepared with a higher molar mass of PEG presented higher permeation features in pure water and larger pores. Water permeation increased with PEG concentration (400 and 600 g/mol). An optimum flux with good solute rejection was achieved at a PEG concentration of ~10 wt%. Additionally, differences in surface morphology and roughness were detected. In another investigation, a polyacrylonitrile (PAN) fibre membrane was used for the removal of chromium (VI) by complexation with hexadecylpyridine chloride [96]. Membranes with 17.5% polymer content were found to retain 98% of the chromium complex (VI), while the remaining ions present in water passed into the permeate. The obtained results confirmed that the complexation–ultrafiltration system may represent an effective method for chromium ion (VI) removal.

### 4.2. Nanofiltration (NF)

The development of the NF membrane began in the mid-1980s, for use in separating small organic molecules and divalent salts positioned between UF and RO. For NF, membranes with a pore size of 1 to 10 nm and MWCO of 200 to 800 Da are commonly used [97]. The application of NF for separating metallic ions is due to the selective thin film structure and small pore sizes. Most of the commercial NF membranes, based on cyclo-aliphatic amine monomers (such as piperazine-PIP), display a negatively charged surface due to the presence of sulfonic and carboxylic groups in the piperazine-amide layer [98]. Aside from commercial membranes, many other modified surface-charged synthetic membranes have also been developed. Zeng et al. developed an innovative NF membrane for Cu (II) removal, comprising PVDF/halloysites nanotubes functionalized with 3-aminopropyltriethoxysilane moieties (A-HNTs)—[99]. The addition of functionalized HNTs significantly improved the metal rejection up to 96.0% compared to the neat membrane. Since the mechanism mostly relied on the Donnan effects, the presence of HNTs also provided access to a larger number of active sites for the co-adsorption of heavy metals. Several studies also reported the performance of layer-by-layer (LbL) membranes to remove metal ions. 

Zhang et al. formed ultrathin graphene oxide (GO) framework layers on hollow fibre support by approaching LbL [100]. First of all, hyper-branched PEI was cross-linked with the membrane surface. To improve its structural stability, the binding sites for the deposition of negatively charged GO nanosheets were prepared through dip-coating, followed by crosslinking with ethylenediamine. An increase in the number of GO nanosheets enhanced the rejection rate of heavy metals, but it had no significant effect on the water flow. Upon removing Pb (II) and Ni (II) from synthetic wastewaters, the recorded rejection rates were 95% and 99%, respectively, at metal concentrations of 1000 ppm at an unfixed pH. This membrane type was also stable, withstanding up to 150 h of continuous filtration of synthetic waters bearing Pb (II) and producing permeate containing Pb (II) lower than the Maximum Contaminant Level (MCL). 

Nayak et al. removed Pb (II), Cd (II), and Cr (VI) from water using surface-modified PVC membranes [101]. To enhance hydrophilicity and metal separation, 4-amino benzoic acid (ABA) was covalently grafted onto the surface of PVC, followed by blending with PS. Under the acidic condition, nearly 100% rejection was achieved for all metals at low concentrations (10 ppm). Instead, only 70–85% rejection rates were recorded for commercial NF membranes (NF 270). The occurrence of ABA’s amino and carboxyl groups yielded better heavy metal rejection.

Zhu and co-workers modified a Thin Film Composite (TFC) hollow fiber membrane by grafting polyamidoamine (PAMAM) onto the polyamide selective layer, recording rejection rates over 99.0% for the Cd (II), Pb (II), Cu (II), and As (V) ions at pH 7.0 and 200 ppm of feed solution [102]. In addition to the decrease in pore size, the amine groups (-NH_2_) in PAMAM led to a positive charge of the membrane, resuming the metals rejection mechanism to steric hindrance and Donnan exclusion. Compared to the UF mixed matrix membranes (MMMs), NF membranes can reject more efficiently a wide range of metal ions. However, its water flux is much lower than the UF membrane. MMMs offer higher water permeability, but their rejection rate at a high metal concentration is often low. Therefore, the NF membrane is more suitable for treating industrial wastewater containing high levels of metal ions. At the same time, UF MMMs are more convenient for treating wastewater containing a low amount of metal ions.

Muthukrishnan and Guha studied the removal of Cr (VI) using different nanofiltration composite polyamide membranes at different concentrations and pH of the membrane feed solution [103]. Two membranes were used for this investigation: (i) a nanofiltration membrane with high rejection (NFI), and (ii) a nanofiltration membrane (NFII) with low rejection. The effect of feed concentration on the rejection percent was found to be relatively low, but the nature of the effect varied with the pH of the solution, with a transition occurring above pH 7.0. The application of NF technologies to treat wastewater containing copper and cadmium ions was also investigated [104]. However, the results show that the RO process led to higher removal efficiencies (98% and 99% for copper and cadmium, respectively), while the removal efficiency of NF was only 90%. Lv et al. investigated an amphoteric polybenzimidazole nanofiltration hollow fibre membrane in terms of cations and anions removal [105]. The molecular weight of the solute, which can be rejected up to 90% by the NF membrane with pore diameters varying from 0.5 to 2 nm, ranged from 200 to 1000 Da [105,106].

Therefore, NF is a promising technology for the rejection of heavy metal ions such as copper [107], nickel [108], arsenic [109,110], and chromium [104], from wastewater. The NF process offers benefits related to the ease of operation, reliability, low energy expenditure, and high pollutant removal efficiency. Figoli et al. (2010) studied the removal of As (V) from synthetic water using two commercial NF membranes (NF90 and N30F), in which it was found that an increase in pH and a decrease in operating temperature and As feed concentration led to higher As removal for both membranes [109]. It was also noted that the feed concentration played a vital role in producing a permeate stream. In recent years, Murthy and Chaudhari devoted much time to study the removal of heavy metal ions using an NF membrane, reporting a thin-film composite polyamide NF membrane for the rejection of nickel ions from aqueous wastewater [110]. The maximum rejection rates recorded for nickel were 98% and 92%, for initial feed concentrations of 5 and 250 mg/L, respectively. The authors have also investigated the capability of a commercial NF membrane to separate binary systems of heavy metals (cadmium and nickel) from aqueous solutions. The maximum solute rejection of nickel and cadmium ions was 98.94% and 82.69%, respectively, for an initial feed concentration of 5 mg/L.

Several theoretical studies have recently predicted that graphene with sub-nanometer-sized pores could act as a highly selective and permeable filtration membrane with higher efficiency, superior thermal stability, mechanical strength, and chemical resistance [56,100,111]. Han et al. reported that ultrathin (22–53 nm thick) graphene NF membranes (uGNMs) on microporous substrates were viable for water purification, attaining high retention rates for organic dyes and moderate retention rates for salt ions [111]. However, the pure water flux of uGNMs was low, 5.0 L m^2^ h^−1^ bar^−1^, when the base-refluxing reduced graphene oxide (brGO) loading exceeded 21.2 mg/m^2^; this being a limiting factor.

Qi et al. developed an innovative NF membrane by grafting polyethylenimine moieties onto the surface of a polysulfone membrane via 2-chloro-1-methyliodopyridine as active agent. This membrane proved to be able to successfully remove divalent and monovalent salts [112].

### 4.3. Microfiltration (MF)

Unlike NF and UF, which are more suitable for removing solutes and small macromolecules, MF is preferred for removing suspended matter. MF is labelled as a low-pressure membrane process. MF membranes can be prepared using both polymers and inorganic precursors [113]. Microfiltration is applied for separating suspended particles with diameters ranging from 0.1 to 10 µm by means of porous membranes [37]. During MF, the static pressure difference acts as a driving force, whereas the separation process relies on the sieving separation. MF occurs under a similar manner as ordinary filtration, but with a higher accuracy. However, when it comes to heavy metals, other approaches are preferred, since MF is more suitable for macromolecular organic compounds and dissolved solids. However, Wang et al. reported the use of such membranes for water loaded with radioactive compounds or heavy metals [114].

### 4.4. Forward Osmosis (FO)

FO can be considered an efficient technology for membrane processes, which involves water transport through a semi-permeable membrane using a natural osmotic process. This process occurs due to the difference of solute concentration found in the Feed Solution (FS, low solute concentration corresponding to low osmotic pressure) and the Draw Solution (DS, high solute concentration corresponding to high osmotic pressure). In the study of Hussein, a commercial asymmetric FO membrane (cellulose triacetate (CTA)) was used for removing Cd ions from water [115,116]. Potassium chloride (KCl) solutions were used for the drawing processes. The study was dedicated to the FO flow and FO batch processes. Both studies were carried out in order to determine the effect of the various parameters upon the Cd removal efficiency. For both analysed processes, it was concluded that water flux may be increased by increasing the concentration of draw solutions (KCl) and the temperature of both feed and draw solutions. It was also observed that the water flux decreased by increasing the operating time of the experiment, the concentration, and pH of feed solution. Overall, the FO flowing process was more efficient than the FO batch process [115,116], with higher rejection efficiency and higher water flux values. 

T. K. Hussein investigated the process of Cd^2+^ ions removal from wastewater using the Forward Osmosis (FO) approach [115]. To meet this purpose, flat sheet membranes were developed using cellulose acetate. Solutions of MgSO_4_·7H_2_0 of different concentrations were used as the DS. For a complete study, the influence of various parameters was investigated according to Table 1, while maintaining a constant pressure of 30 bar gauge.

It was concluded that flat sheet CA membranes are suitable for Cd ions removal. In this respect, the water flux permeates increased when the concentration of the MgSO_4_·7H_2_O draw solutions, the temperature of both the feed and draw solution, and the flow rate of the feed solution were higher. It was also noticed that increasing the Cd^2+^ ions concentration in the feed solution, the duration of the experiment, and the flow rate of draw solutions can lead to a decrease of the water flux. Additionally, the concentration of Cd^2+^ ions in the outlet of the feed solution increased with the concentration of the feed and draw solution, the experiment run time, the flow rate of the feed solution, and the temperature of feed and draw solutions, while it decreased by increasing the draw solution flow rate. The reverse salts flux of MgSO_4_·7H_2_O through the CA membrane decreased in time. The CA membrane’s removal efficiency (R %) decreased with time, reaching 78.87% after 3 h [117]. FO using a CTA membrane was also performed for evaluating the zinc ions removal efficiency. This study focused on investigating the effect of the draw solution concentration (10–150 g/L), the pH of the feed solution (4–7), and the feed solution concentration (10–100 mg/L), in which case a removal efficiency of 96.2% was achieved [117].

### 4.5. Reverse Osmosis (RO)

**Figure 5:**
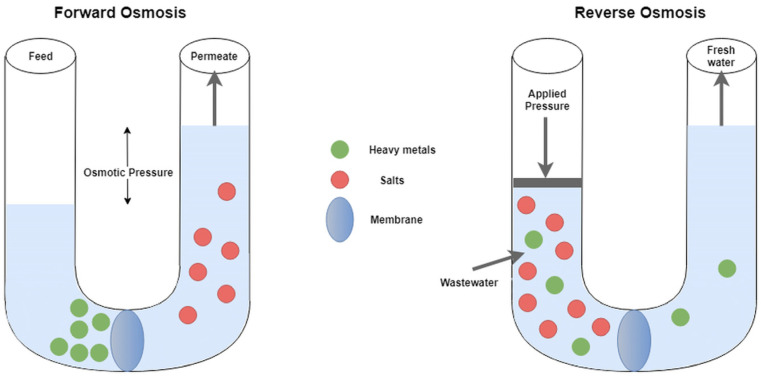
FO vs. RO, exemplified for different classes of pollutants.

Osmosis may be simply defined as the spontaneous movement of a solvent across a semipermeable membrane from a less concentrated to a more concentrated solution. The ideal semipermeable membrane does not allow solute movement, only solvent flow [118]. Reverse Osmosis (RO) is a separation process carried out using semi-permeable membranes, which occurs upon applying pressure. During RO processes, the solute is retained on one side, whereas the solvent passes through the other side. The RO process involves forcing a solvent from a region of high solute concentration through a membrane into an area of low solute concentration by applying pressure above the osmotic pressure. It is called RO as it is the reverse of normal osmotic pressure (i.e., FO; for a better understanding, both processes are shown in Figure 5), which consists of the natural process of solvent movement from an area of low solute concentration through an area of high solute concentration without any external pressure. Therefore, it may be stated that RO is a pressure-driven process.

The used membranes for RO processes exhibit a dense barrier layer in the polymer matrix, where most of the separation takes place. Usually, the membrane allows only water to pass through this dense layer and prevents the passage of dissolved substances (such as salt ions). For this process to occur, high pressure must be exerted on the high concentration side of the membrane [119,120]. Among the areas of RO use, it is worth mentioning water recycling processes and metal recovery purposes [121]. RO proved to be the easiest approach of desalting seawater and brackish water. Cellulose acetate is a widely used polymer for RO membranes [115].

The interest in using RO in water recycling also allows for the removal of several types of molecules and ions, providing potable water. RO is suitable for different water purification operations, including desalination of the bulk of dissolved salts in wastewaters (WW) rising from various industry branches. This widespread use of RO can be attributed to the fact that it is suitable for the removal of both organic and inorganic pollutants, but also suspended solids and high molecular weight compounds [122].

Literature studies deal with removing heavy metals by means of different types of RO membranes, and the effects of different working parameters are being investigated [121,122,123]. Furthermore, one study compared RO and NF. Another study looked at the need for pre-treatment. The literature describes the use of both commercial and synthetic RO polymeric membranes. In this context, it should be mentioned the use of polyamide thin-film composite membrane commercialized under the trade name TW30-1812-50, for the removal of Ni, Cu, and Zn [121,123], synthetic water being used for this purpose (containing CuSO_4_·5H_2_O, Cu(NO_3_)_2_·3H_2_O, Ni(NO_3_)_2_·6H_2_O, and ZnSO_4_·7H_2_O). In this case, the permeate flux decreased with increasing concentration. A significant difference in flux decline between copper nitrate and copper sulphate was observed, which enabled establishing the influence of the anions. It was also shown that the heavy metal concentration significantly influenced the membrane separation process. At a low concentration of heavy metals, the permeate flux decreased significantly, and a higher concentration of heavy metals led to a linear decline. Additionally, a very low influence upon the flux was recorded with the increase of heavy metal concentration at low transmembrane pressures. The experiments reveal that transmembrane pressure plays an essential role in membrane separation processes [122].

Ventura et al. investigated the efficiency of commercial membranes (polyamides-PA and cellulose acetate-CA) in the Cd removal process [124]. Cellulose acetate, i.e., cellulose esters, is helpful in a wide range of domains but primarily for coatings, films, and membranes. For this purpose, synthetic solutions of cadmium sulphate at concentrations of 150 and 500 mg L^−1^ were prepared. Both RO and NF membrane treatments proved capable of achieving advanced cadmium sulphate removal. Even though Nanofiltration (membrane NF-90-PA) gives higher hydraulic permeability due to its nanopores and the convective transport, the average degree of rejection of Cd^2+^ ions was not higher than the one observed in RO processes. Therefore, the best results in removing cadmium sulphate were obtained using the cellulose acetate RO membrane (HRP98PP-CA) and the low-energy reverse osmosis membrane (BW30LE-PA). These membranes provided an excellent degree of ion rejection and elevated water fluxes under operating conditions. However, the BW30LE-PA offers more advantages than the CA membranes, as the latter is biodegradable, has low chemical resistance to acid solutions, and uses little pressure to operate and, thus, lower operational costs.

Ates et al. investigated the decontamination process of waste waters (WW) rising from the aluminium industry through UF, NF, and RO, as these WW proved to pose an environmental impact given their harsh pH, high load in heavy metals, and conductivity [125]. The following membranes were used in the experiments: polyether sulfone- PTUF (ultrafiltration), an NF270 semi-aromatic piperazine-based PA layer on top of a polysulfone microporous support reinforced with a polyester nonwoven backing layer (nanofiltration), and RO membranes SW30 with MWCO 5000, 200–400, and 100 Da, respectively. When using FeCl_3_ and FeSO_4_ within precipitation experiments, lower removal performance was achieved for aluminium and chromium, below 35%, when operating at ambient temperature with wastewater of pH ~3. Experimental results of membrane filtration tests show that both NF and RO membranes can effectively remove aluminium, total chromium, and nickel (>90%) from the aluminium production wastewater. The RO membrane (SW30) revealed a slightly better performance at 20 bar operating pressure in terms of conductivity removal values (90%) compared to the NF270 membrane (87%). Although similar performances of heavy metals removal and conductivity were observed for NF270 and SW30, significantly higher fluxes were obtained for NF270 membrane filtration at any pressure, exceedingly more than three times the flux values in SW30 membrane filtration. Due to the lower content in heavy metals (<65%) and lower conductivity (<30%), it was assumed that pre-treatment followed by NF may represent a viable solution for protecting and extending the lifetime of the NF membrane. The water treated by both NF and RO can be recycled back into the process for reuse with economic and environmental benefits [124].

Thaci et al. investigated the pre-treatment process with biowastes before RO (with cellulose acetate coal asymmetric membranes) [126]. Therefore, biowastes (wheat bran, corn cobs, and olive waste) were involved in the adsorption process of heavy metals from wastewaters. It was found that short contact time, pH, and temperature are very convenient for further treatment of industrial wastewaters by RO without adding an agent. The use of bio-sorbents was beneficial for the applicability of heterogeneous asymmetric RO membranes. The investigated bio-sorbents could be feasible and sustainable materials for pre-treating wastewater (bearing Pb, Cd, Ni, Zn, Mn, Co) before treatment by RO means. Combining these methods, suitable premises for the complete recovery of heavy metals from water and wastewater, water recovery, and reuse may be created [112].

Selimi et al. investigated the process of separating heavy metal salts dissolved in water through RO. For this purpose, heterogeneous RO membranes of coal-modified cellulose acetate (317K-2) were used. Selectivity properties in RO membranes were investigated using various feed solutions, such as aqueous solutions of HgCl_2_, KMnO_4_, CrCl_3_, and CuSO_4_. The composition of cellulose acetate and powdered coal in the ratio of 1:1.75 (by weight) was investigated in the casting solvent (acetone), and casting conditions were analysed. Powdered coal was chemically modified using the 4-nitrobenzene diazonium salt. Similar to cellulose acetate membranes, the cellulose acetate coal-modified membranes separate trivalent and bivalent ions more than univalent ions. Interest in RO was given due to the fact that it allows the separation not only of organic compounds, but also of inorganic ones from wastewater. In this respect, the 317K-2 membranes prepared from cellulose acetate and coal modified with a 4-nitrobenzene diazonium salt in acetonitrile revealed improved performance compared to cellulose acetate and unmodified coal (316K-2). For those heterogenous batches of cellulose acetate–coal membranes, the 317K-2 membranes showed better performance than 316K-2 membranes in separating inorganic salts solutions. The separation sequence was CuSO_4_ > KMnO_4_ > CrCl_3_ > HgCl_2_ > NaCl. Based on the obtained result for solute separation, the membranes 317K-2 were found to be suitable for the separation of inorganic salts solutions, which occur in the natural system and wastewater [127].

Kamaruzaman obtained Cellulose Acetate Membranes (CAM) and investigated their performance in removing heavy metals. For a complete study, the prepared membranes were fully characterized using various modern techniques: Field Emission Scanning Electron Microscopy (FESEM), BET surface area (BET), and Fourier Transform Infrared (FT-IR) spectrometry. The CAM adsorption features were investigated for Cu (II) and Cd (II) ions. The effects of pH, adsorbent dosage, initial metal concentration, kinetic parameter, desorption, and reusability on CAM adsorption capacity were investigated in a batch adsorption mode. The membranes act as adsorbents as they carry active sites (OCOCH_3_ and –OH), where Cd (II) and Cu (II) ions interact. FTIR spectra confirmed the formation of CAM as characteristic peaks occured at 1732 cm^−1^ due to C=O vibrational modes after casting, and the C−H and CO– stretching bands were detected at 1371 cm^−1^ and 1227 cm^−1^, respectively. Experimental adsorption results indicate that a higher initial pH value corresponds to a higher adsorption capacity. The optimum adsorption condition of CAM against Cu (II) and Cd (II) ions occurred at pH 8 and pH 10 with adsorption capacities of 14.22 mg/g and 11.20 mg/g, respectively. Moreover, 0.035 g and 0.012 g of adsorbent were optimal for the adsorption of Cu (II) and Cd (II) ions, corresponding to the maximum adsorption efficiencies with a removal percentage of about 79.89% and 76.62%, respectively. The maximum sorption capacities calculated from the Freundlich isotherm were 0.93 for Cu (II) and 1.30 mg/g for Cd (II) ions. A kinetic study on the sorption of Cu (II) and Cd (II) ions by CAM showed a pseudo-second-order kinetic pattern, and the rate constant K2 was 0.66 and1.75 g/mg min, respectively. In the desorption study, the highest desorption percentage of Cu (II) and Cd (II) ions by CAM, i.e., 32.06% and 44.21%, respectively, was achieved with 1 M sulfuric acid and hydrochloric acid solution. When three adsorption experimental cycles were conducted, the adsorption capacity did not change significantly, thus confirming their reusability. In conclusion, it was found that CAM is efficient for removing Cu (II) and Cd (II) ions from real environmental water samples obtained from rivers, seas, lakes, and drinking water [128].

Al-Alawy et al. investigated the removal of heavy metals using synthetic wastewaters carrying Zn, Ni, Cu, and Cr. A spiral sheathing of polyamide was used for NF and RO purposes to compare these approaches. The polyamide NF membrane allowed chromium and copper ions to permeate to acceptable limits. The polyamide RO membrane proved to be highly efficient for the removal of chromium, copper, nickel, and zinc ions, in which case it also allowed for a lower permeation of these ions than the admissible limits. The rejection in the first three minutes when the feed concentration was approximately constant for chromium in NF and RO was 99.7% and 99.93%, for copper was 98.43% and 99.33%, for zinc was 97.96% and 99.49%, and for nickel was 97.18% and 99.49%, respectively. The maximum recovery for chromium in NF and RO was 71.75% and 48.5%, for copper was 75.62% and 50.68%, for zinc was 80.87% and 54.56%, and for nickel was 60.06% and 46.18%, respectively. For a mixture of synthetic electroplating wastewater, NF and RO membranes revealed a high rejection percentage for heavy metal ions [129]. In Table 2, literature information on heavy metals removal using RO is summarized.

### 4.6. Electrodialysis (ED)

**Figure 6:**
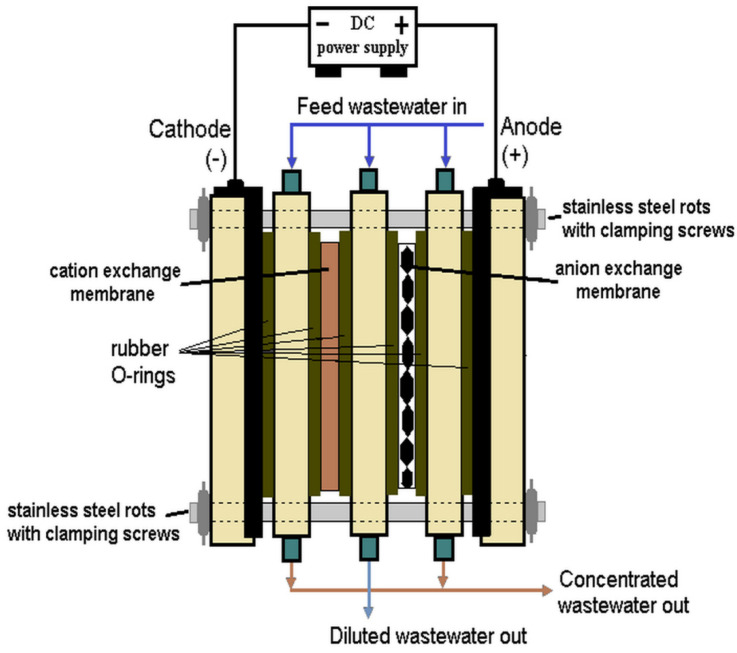
Schematic representation of the ED cell with three compartments.

ED may be defined as an electrochemical separation method. During ED, positively charged heavy metals move to the corresponding electrodes through a cation-exchange membrane. Under the influence of an electrical potential difference used as a driving force, the anions migrate toward the anode and the cations toward the cathode, crossing the anion-exchange membrane and cation-exchange membrane, respectively, to recover the metal [89,130,131,132,133,134,135]. In the literature, many electrodialysis systems are indicated with three or more wastewater treatment compartments [134,136,137,138,139,140]. An ED cell with three compartments and ion-exchange membranes is schematically shown in Figure 6.

Compared to other processes (i.e., UF, RO, NF, electro deionization) used for the treatment of heavy metal laden wastewater, ED has many advantages, such as the capacity to produce a highly concentrated stream for recovery and the rejection of unwanted impurities from water, no regeneration reagents are required to make the conventional ion exchange process, and feasibility in removing heavy metals at a low concentration. Additionally, ED is environmentally friendly, convenient to operate, has low energy consumption, and requires minimal investment costs for specific feed and product water compositions. Besides these advantages, ED has several disadvantages, such as: in the process of potable water production, only ions are removed, meaning uncharged components, such as microorganisms or organic contaminants, will not be removed; there is relatively high energy consumption when solutions with high salt concentrations must be processed; the investment costs are relatively high when in the dilute compartment the salt concentrations must be very low due to the low limiting current density, which requires a large membrane area [135,136,141].

The effect of different factors/parameters (feed solution pH, flow rate, temperature, current density, voltage, concentration, concentration polarization, membrane type, membrane properties, and cell compartments geometry) on ED performance has been examined in several studies [43,134,142,143,144,145]. The solution pH can affect the distribution of ions between the charged sites on the membrane surfaces [141]. It has also been reported that the pH control in the dilute compartment is essential and can be adjusted to increase the current efficiency of the removal of ions [138]. The performance of the ED cell was enhanced by the increase of the voltage and the temperature, but a decrease in the separation percentage occurred when increasing the flow rate [144,146].

Ho Choi and Jeoung reported that the applied voltage, the flow velocity, and the dilute solution’s initial concentration may be considered the main parameters influencing energy consumption [138]. The current density is an important operating parameter of the ED process. Operation at high current densities without measuring the limiting current density can increase energy consumption, which is detrimental to the membrane [132]. The concentration polarization occurs in all membrane separation processes. In an ED cell, the concentration polarization leads to an accumulation of ions on the membrane surface, leading to salt precipitation when the concentration exceeds the salt’s solubility limit. Also, membrane fouling is considered a significant problem affecting almost all membrane separation processes [43,141].

ED has been applied in numerous domains, such as seawater and brackish water desalination, wastewater treatment [147,148,149,150], fermentation broth desalination [151], metal ion separation [133,151], food processing [152], and dye removal [138,153]. ED with ion-exchange membranes has been successfully used for the recovery/removal of different heavy metals (i.e., copper, lead, nickel, zinc, chromium, cobalt, manganese, mercury, antimony, iron) from other wastewaters. Table 3 summarizes the literature information on extraction percentages of different metallic ions from wastewaters using ED.

Abou-Shady et al. investigated the effect of pH on ED separation of Pb^2+^ and NO_3_^−^ in terms of concentration ratio, concentration polarization, current efficiency, and energy consumption [141]. The experiments were carried out using an ED unit consisting of two membrane stacks positioned between three graphite electrodes, each membrane stack containing 55 pairs. The results show that the energy consumption was higher due to the increase in current associated with low pH; the maximum being almost 2.5 Wh L^−1^ at 60 V and pH 2.5. At a pH between 2.5–5 and 10 V, the energy consumption was less than 0.25 Wh L^−1^. Also, they reported that the Pb^2+^ concentrations in the dilute solution varied between 48 and 55 mg L^−1^, corresponding to a 93.1–94% removal efficiency.

Chapotot et al. investigated the differences between the transport of monovalent and divalent cations across cation exchange membranes [152]. They reported that the copper transport rates of 0.4–2.5 mol h^−1 ^m^−2^ and the sulphate transport rates of 10–16 mol h^−1^ m^−2^ were obtained for an applied current density of 500 Am^−2^ and an electrolyte flow rate of 60 L h^−1^. Cifuentes et al. evaluated the electrodialysis cell performance of aqueous sulfuric acid–copper sulphate solutions, with and without recirculation [153]. They also studied the presence of impurities such as arsenic (As) and antimony (Sb), typical of copper electrorefining electrolytes. The experimental results show that the concentrations were 3–9 g L^−1^ copper, 50 g L^−1^ sulphuric acid, 3 g L^−1^ arsenic, and 0.025 g L^−1^ antimony. At 22 °C and 44 °C, the membrane transport rates were 0.2–0.6 mol h^−1^ m^−2^ for copper, 0.65–2.8 mol h^−1^ m^−2^ for sulphate, and 0.016–0.03 mol h^−1^ m^−2^ for arsenic. Therefore, the electrodialysis system was successfully applied to the separation and concentration of chemical species.

Santorosa et al. developed a mini-ED cell with an acrylic two-compartment configuration with parallel Platin electrodes and different cationic membranes (Nafion 417 and MZA). The system was used for the Zn^2+^/Ni^2+^ separation gradient and ion-selective membrane analysis at a current density of 40 mA cm^−2^. The authors concluded that the most significant degree (90%) of recovered Zn and Ni ions occurred five hours after industrial effluent processing [154].

Chaudhary et al. reported the optimum conditions for separating nickel impurities from cobalt solutions by electrodialysis, exploiting the superior stability of the EDTA complex with nickel [149]. They reported that most of the cobalt ions would remain in the solution in the catholyte chamber, but the metal could be deposited on the cathode by increasing the pH to 4–4.5. The optimal conditions for a complete removal of Ni from cobalt sulphate solutions were an EDTA: Ni mole ratio of 1:0.85, a sulfuric acid concentration of 0.03 ± 0.05 mol dm^−3^ in the feed chamber, and a current density of 41.5 A m^−2^.

Abo-Ghander et al. developed a modified cell that integrated ED and demonstrated that the cell could recover metallic copper from wastewater containing 1000 ppm cupric ions and reduce the concentration to about 1 ppm [142]. Căprărescu et al. have investigated the efficiency of removing Zn (II) ions from simulated wastewaters by a mini-electrodialysis system and an adsorbents/ion exchanges membrane [132]. The system was operated at constant voltage (5 V) using different concentrations of synthetic zinc ion solutions (1.5, 3, and 6 g/L) with and without electrolyte recirculation for 1.5 h. The flow rate was 6.6 mL/min. The results show that without electrolyte recirculation, the zinc ions removal ratio was higher (>70%) when higher concentration solutions were treated (6 g/L). When the solution was recirculated at a higher concentration (6 g/L), the flux of zinc ions through the membranes and energy consumption were higher (39.59 g/m^2^ h; 242.73 kWh/m^3^). In another study, the removal of copper ions from synthetic electroplating wastewater was performed by applying electrodialysis with heterogeneous membranes [134]. The highest extraction degree (over 70%) was recorded under potentiostatic control at 8 V, and for an electrodialysis time of 90 min.

Ho Choi and Jeoung investigated the effects of different operating parameters, such as the initial dilute solution concentration, the flow velocity, and the applied voltage, on the removal rate of Zn^2+^ in the model solution using an electrodialysis system with CMX cation exchange membranes and AMX anion exchange membranes [138]. They reported that an increase in the initial concentration of the dilute solution in the flow velocity and in the applied voltage resulted in an increase in the dialytic rate and in the maximum current value. The results show that the energy consumption increased (removal ratio = 90%) due to an increase in the initial concentration of the dilute solution and applied voltage, respectively.

Mohammadi et al. studied the separation of copper ions from a solution using a laboratory electrodialysis set-up and two types of ion-exchange membranes [155]. They applied Taguchi’s method to determine the optimal operating conditions of the experiment and studied four parameters at three levels: concentration (100, 500, 1000 ppm), temperature (25, 40, 60 °C), flow rate (0.07, 0.7, 1.2 mL/s), and voltage (10, 20, 30 V). Thus, the results reveal that the optimal levels were a concentration of 1000 ppm, a temperature of 60 °C, a flow rate of 0.07 mL/s, and a voltage of 30 V. It was also found that the use of a pair of membranes with a higher ion-exchange capacity improves performance and the removal percentage was 94.94% and 97.33%, respectively, for the membranes used. In another study by this group, the effects of operating parameters on the separation of lead from wastewater using electrodialysis and two commercial membranes were investigated [156]. The results showed that increasing temperature and voltage improves electrodialysis cell performance, but the separation percentage decreases when the flow rate increases. However, the separation percentage was 63.29% for membrane type 1 and 71.01% for membrane type 2, respectively.

Metal finishing wastewater containing ions of Zn, Ni, Cu, Fe, and Al was treated by electrodialysis with Nafion and Selemion membranes [135]. The extraction percentage of different metal ions after 6 h of treatment in the electrodialysis pilot plant were found to be 20.3%, 24.1%, 14.2%, 29.7%, and 77.4%, respectively when performed under potentiostatic control, and 68.5%, −9.6%, 23.5%, 46.0%, and 46.7%, respectively, when performed under galvanostatic control. The authors reported that the anomalous behaviour of the Fe ions can be attributed to the presence of a relatively large amount of undissociated iron coordination complexes, such as Fe[Fe(CN)_6_].

Ibáñez et al. studied aqueous acidic solutions of copper, arsenic, and antimony using commercial ion exchange membranes in a batch electrodialysis cell [139]. They reported that the transport rate of copper through the cation exchange membrane was 0.5 and 0.75 mol/h/m^2^ for 150 and 225 A/m^2^, respectively. Also, in a 1.5 mol/L acidic solution, As (III) was transported as AsO^+^ at a transport rate of 0.02 mol/h/m^2^ at a current density of 225 A/m^2^, and As(V) was transported as H_2_AsO_4^−^_ at a transport rate of 0.01 mol/h/m^2^ in 0.5 mol/L of acid. Antimony was transported across both anion and cation membranes at 2.7 and 3.4 mmol/h/m^2^.

Öğütveren et al. studied the feasibility of copper removal from wastewater using a recirculating electrodialysis system made of Perspex sheet with graphite anode and a stainless steel cathode [157]. The cell was separated by an anion exchange membrane (IonacMA3475) and a cation exchange membrane (Nafion423). They reported that a 91% removal rate was achieved at 20 V with an energy consumption of 17.01 kWh/m^3^. In contrast, a removal rate of 99.9% was achieved with an energy consumption of 65.0 kWh/m^3^ at 40 V in 75 min, for an initial concentration of 100 mg Cu^2+^/L water.

A detailed study on the transfer behaviour of Mn (II) through ion-exchange membranes performed in the electrodialysis device, under the galvanostatic mode, was reported by Melnyk et al. [158]. The results indicate that an increase in the concentration of sulphate ions in water under electrodialysis conditions decreases the formation of poorly soluble manganese compounds on membranes.

Even though many polymers may be involved in membrane development, only a few gained practical significance [159]. The addition of PANI to PAN nanofiber membranes was found to endow them with improved adsorbent properties over lead and chromate ions [160].

Y. Liu et al. described the use of a bipolar membrane ED system [161]. The developed system consists of three compartments: (i) anode compartment; (ii) chitosan compartment; and (iii) heavy metal (cathode) compartment. Therefore, a three-step method was approached, which produced insignificant results, since only small amounts of residual metals remained in chitosan. This method may be also approached to remove heavy metals from other solid wastes.

Chan et al. applied the ED process to recover valuable metals from postconsumer lithium-ion batteries [162]. Prior to electrodialysis, a chelation is carried out by means of EDTA. In this way, not only can the pollution effect be diminished, but a secondary source of strategic materials may also be provided.

ED may be used in combination with other approaches, resulting in a synergy of benefits. For instance, Yan et al. combined electrodialysis and electrodeposition for treating the spent electroless nickel plating bath. In the study, a thorough investigation was carried out to determine the effect of all the parameters involved (current density, operation time, pH value of the spent bath, dilution multiple, flow rate, and electrolyte (Na_2_SO_4_) concentration) on the recovery, removal rate, and energy consumption. It was found that not only were Ni^2+^ ions efficiently removed, but removal of HPO_3_^2−^ ions was achieved simultaneously [163].

The opposite process, i.e., reverse electro-dialysis (RED) can be also promoted. A particular case of RED is bipolar membrane reverse electrolysis, abbreviated BMRED. In brief, BMED relies on the finding that water molecules existing in the intermediate layer of bipolar membranes can split into H^+^ and HO^−^ ions. Similarly, BMED may achieve the transformation of salts into related acids and alkalis [36].

### 4.7. Chemical Modification with the Aim of Attaining Improved Properties

It was found that under certain situations, the performance of polymer membranes by themselves is not satisfactory. Therefore, chemical modifications were approached involving mixing with a second polymer, covering with a polymer layer, or use of inorganic additives. For instance, inexpensive adsorbents, such as carbon powders of either natural or synthetic origin, may be loaded within polymer membranes for improving the adsorbent features, along with additional control of the final properties by varying the amount of used powder [134]. Moreover, polymer coating and blending brings about the possibility of overcoming the mechanical stability issues of pure polymer systems. In this respect, El- Gendi et al. reported the use of Na_2_EDTA as an eluent for PA-6/H_2_O membranes [13].

The rejected species are also prone to accumulate at membrane surface, causing its fouling. In this context, it becomes worth mentioning that the fouling resistance of P84-PEI membranes (developed by using P84 commercial powder along with polyetherimide- PI) may be well enhanced by covering them with polyvinyl alcohol (PVA) [97]. For example, Zheng et al. developed a positively charged P84 NF membrane using a commercial powder P84 with a molecular weight of 153,000 g/mol, as a precursor. This membrane was crosslinked, under mild conditions, with PEI. This crosslinking process endowed the membrane with a better ability to reject organic dyes. Moreover, this membrane was also able to act on divalent heavy metals [164].

Natural polymers, such as chitosan and carboxymethyl cellulose, can also be used in the development of ultrafiltration membranes [165]. Literature studies reveal that mixing polymers with chitosan is beneficial not only for the removal efficiency, which is enhanced due to chitosan porosity, but fouling issues are somewhat mitigated [166].

The use of various inorganic compounds has also proved to be beneficial. Studies have indicated that integrating inorganic particles into membranes lead to Mixed Matrix Membranes (MMM) [167]. In this case, magnetite, carbon nanotubes, or other carbon adsorbent materials provide enhanced features of the developed membrane [126]. Among other materials that were shown to improve decontamination performances and/or membrane features successfully, it is worth mentioning: carbon nanotubes [125], biochars and bio-sorbents [168], compounds with biocide action (TiO_2_), clays, silicons, graphene oxide, powdered coal [117,127,169,170]. At the same time, cheap materials, such as bauxite residue (red mud), can be used for this purpose, due to their high content of metal oxides [162]. Moreover, when using chitosan as a precursor for membranes, mechanical properties issues arise, which may be well surpassed upon the addition of a wide range of inorganic additives, which include alumina, titania, iron oxide, magnetite, and montmorillonite. Besides, the use of these additives also endows the membranes with adsorptive features [171].

Carbonaceous materials can be developed using low-cost agricultural wastes, such as tobacco leaves, apricot stones, or biomass-derived carbon powders [172]. Another carbonaceous material that was reported in literature to enhance HMs removal efficiency is graphene oxide [173].

**Table 3:** The values of extraction percentage of different metallic ions from various wastewaters using electrodialysis.

Metallic Ion	Type of Membrane	Optimal Operational Conditions	Maximum Percentage Extraction (%)	Ref.
Zn	adsorbents/ion exchange resins-PVA membranes	voltage of 5 V, 1.5 h	80.11	[132]
Zn	organically modified montmorillonite polyethersulfone membranes	voltage of 5 V, 1 h	~95	[133]
Mg	ion exchange membranes	current density of 1.65 mA cm^−2^, 930 h	84	[135]
Cu	styrene-acrylonitrile copolymer membrane	voltage of 5 V, 1.5 h	70.31	[136]
Zn, Cu, Ni Al	Nafion 450 and Selemion membranes	current density of 10 mA cm^−2^, 6 h voltage of 5 V, 6 h	68.5, 23.5, 46.0 77.4	[137]
Zn	ion exchange membranes	voltage of 13 V, 25 min, flow velocity of 8 cm s^−1^	99.35	[138]
Cu, As, Sb	ion exchange membranes	current density of 225 A m^−2^, 3 h	68, 78, 85	[139]
Pb, Cr Cu, Zn	(AR204SXR412) and (CR67, MK111) anion and cation exchange membranes AMV and CMV anion and cation exchange membranes	voltage of 30 V	38.68, 39.00 41.52, 43.88	[140]
Zn, Ni	Nafion 417 commercial membranes	current density of 40 mA cm^−2^, 2 h	90	[154]
As, Cd, Co, Cr, Cu, Fe, Mg, Ni, Zn	bipolar membrane	current density of 3.0 mA cm^−2^, 45 h	93.4, 90.9, 71.5, 69.5, 98.8, 94.8, 91.5, 75.7, 96.4	[161]
Fe	membrane containing cellulose acetate and chitosan-silver ions	voltage of 15 V, 1 h	63.7	[174]

Căprărescu et al. modified PVA-based membranes with rosehip extract and used them for the removal of crystal violet dye [144]. This same group also developed a biopolymeric ED membrane with metal ions retention features starting from a hybrid system, i.e., cellulose acetate/chitosan- AgNO_3_/PEG [174].

Peydayesh et al. elaborated a hybrid system by self-assembling ethylenediamine-grafted multiwalled carbon nanotubes (ED-g-MWCNT) on the top layer of a PES membrane [175].

Aside from using various additives/fillers, modification of membranes can be also carried out by using specific chemical reagents that endow the membrane with new functional groups. In this context, it is worth noting that Mu et al. described the enhancement of the membrane’s final properties (PWF and inorganic salts rejection behaviour) upon its modification with quaternary ammonium moieties [176]. Yang et al. approached nonsolvent-induced phase separation to yield mixed matrix PVC-based membranes. To this end, poly (methyl methacrylate-co-dimethylaminoethyl methacrylate) (PMD) was used along with PVC. This hybrid membrane largely removed traces of heavy metal ions [177]. Other studies reported that regenerated cellulose grafted with glycidyl methacrylate (GMA) and functionalized with imidazole groups (cellulose-g-GMA-imidazole) revealed adsorbent efficiency for Pb and Ni ions [129].

The addition of certain additives may also significantly enhance the rejection process of heavy metals. In this case, it was found that the addition of a curcumin-modified boehmite to a polysulfone membrane resulted in an efficient removal of a wide range of metal cations (Fe^2+^, Cu^2+^, Pb^2+^, Mn^2+^, Ni^2+^, and Zn^2+^) [178].

Kamari and Shahbazi developed a biocomposite by modifying chitosan with SiO_2_·Fe_3_O_4_, further used as an NF membranes precursor. The membrane prepared in this way proved capable of simultaneously removing salts, dyes, and heavy metals [179].

Nayak et al. proposed a four-component system, i.e., polyphenylsulfone/multiwalled carbon nanotubes/polyvinylpyrrolidone/1-methyl-2-pyrrolidone, as a precursor for UF membranes, suitable for heavy metals removal [180].

## 5. Conclusions

The world currently faces important water pollution issues, primarily due to industrial development. HMs are among the most harmful contaminants, mainly because they are not biodegradable. Therefore, much research has been aimed at reducing their impact. Of all the known processes for removing heavy metals, membrane processes have gained particular importance, becoming an important field of study. The interest in membranes is caused by the flexibility of the approach and of the precursor material (ceramics, polymers, and more). Additionally, both laboratory-synthesized and commercially available membranes may be used. An example of a commercial membrane is a thin-film composite polyamide nanofiltration membrane (under the trade name AFC 80) [38,39]. Another important aspect in membrane processes is the type of membranes available. In this respect, special attention was paid to synthetic polymers, as the final properties of the membranes can be tailored through the preparation conditions. Additional control is also possible by using mixtures of polymers instead of single polymer systems.

NF polymer membranes have proved to be efficient for the removal of various heavy metals, including Cu, Pb, Cr (VI), and Cd. Developing hybrid systems by using inorganic carbonaceous materials, such as HNTs or GO, together with polymers, can improve NF performance. Although UF benefits from low operating pressure, high permeation flux, low operating cost, and scalability, it fails in removing of harmful ions and small molecules. This is why UF is used mainly as Micellar Enhanced Ultrafiltration (MEUF) and Polymer Enhanced Ultrafiltration (PEUF) for heavy metals retention. On the other hand, RO can remove a wide range of contaminants, such as heavy metals, oily effluents, salt ions, and organic wastes, for producing drinking water. It also appears that cellulose acetate (CA)-based RO membranes are able to accomplish an advanced removal of Cd (II), Pb, Ni, and Cu (II). Nevertheless, improvements may be achieved using biowastes-derived coal as an additive or by grafting imidazole groups. Finally, ED can be used for HMs retention and for desalination. However, unlike RO membranes, desalination by ED is achievable with good results only for very diluted solutions. Purolite and Nafion commercial membranes are the most used in ED processes.

Several studies have also reported that the efficiency of membranes can be improved by integrating a wide range of inorganic compounds and additives (CNTs, magnetite, GO, and carbon powders) in membrane preparation or by chemically modifying the polymers functional groups. A particular development is expected for such membranes with adsorbent properties, also known as adsorptive membranes, which are obtained by combining polymers and adsorbent powders. Because of their affinity towards ions and molecules, adsorptive membranes are also referred to as affinity membranes. The adsorbent component may be added to polymer membranes via three approaches: grafting, blending, and assembling. Nevertheless, there is a long wait until such new technologies will be available for wastewater purification in water treatment plants. For this reason, this review serves as a starting point for future studies on the treatment of different wastewaters for HMs removal. The information provided herein is relevant for the scientific community dealing with restoring water quality by membranes technologies, as well as for the various industry actors, as support for preventing the emission of hazardous materials into surface water bodies or groundwater.

## Figures and Tables

**Table 1:** Values of process parameters [115].

Investigated Parameter	Range
Concentration of draw solution	10–150 g/L
Concentration of feed solution	10–200 mg/L
Flow rate of draw solution	30–100 L/hr
Flow rate of feed solution	30–100 L/hr
Temperature of draw solution	10–40 °C
Temperature of feed solution	10–40 °C

**Table 2:** Reported membranes and their use.

No.	Water That Needs to Be Treated	Removed Heavy Metal	Rejection Percentage (%)	Membrane Used	Work
1.	Drinking water supplies	Ni, Cu, Zn	-	PA—TW-30-1812-50	[118]
2.	Industrial processes in metal mechanics	Cd	>95 >95 >97 >90	HRP98PP-CA SW 30-PA BW30 LE-PA NF-90-PA	[124]
3.	Aluminium oxidation wastewater	WW from the aluminium industry (Al, Cr, Ni)	87 90	PTUF NF270 SW 30	[125]
4.	Mining flotation process effluents	Pb, Cd, Ni, Zn, Mn, Co	>95%	Biowastes for pre-treatment + cellulose acetate—coal asymmetric RO membrane	[126]
5.	Synthetic wastewaters; mining flotation process	Hg, Mn, Cr, Cu	-	Coal modified cellulose acetate: 317K-2 316K-2	[127]
6.	Steel manufacturing plant	Cu, Cd	79.89; 76.62	Cellulose Acetate Membrane (CAM)	[128]
7.	Steel manufacturing plant	Zn, Ni, Cu, Cr	99.49; 99.49; 99.33; 99.93	Polyamide membranes	[129]

## Data Availability

Not applicable.
